# Melioidosis in Brunei Darussalam

**DOI:** 10.3390/tropicalmed3010020

**Published:** 2018-02-19

**Authors:** Ketan Pande, Khairul Azmi Abd Kadir, Rosmonaliza Asli, Vui Heng Chong

**Affiliations:** 1 Department of Orthopaedics, Raja Isteri Pengiran Anak Saleha Hospital, Bandar Seri Begawan BG 1710, Negara Brunei Darussalam; khairulazmiabdkadir@gmail.com; 2 Department of Medicine, Raja Isteri Pengiran Anak Saleha Hospital, Bandar Seri Begawan BG 1710, Negara Brunei Darussalam; ROSMONALIZA.ASLI@moh.gov.bn (R.A.); vuiheng.chong@moh.gov.bn (V.H.C.)

**Keywords:** melioidosis, *Burkholderia pseudomallei*, epidemiology, diagnosis, treatment

## Abstract

Melioidosis continues to be a major health care problem in Brunei Darussalam. The age of patients, gender distribution, risk factors, and clinical presentations are similar to those reported from other countries in the region. The incidence of melioidosis was high during the wet months and in the Temburong district, which has the highest annual rainfall. In spite of adequate facilities for diagnosis and treatment, the mortality remains high (27%). Women and those presenting with septic shock had higher mortality. There is a case for making melioidosis a notifiable disease in Brunei Darussalam. Coordinated efforts between policy-makers and various stakeholders are required to effectively combat the disease.

## 1. Introduction

Melioidosis is endemic in many countries of Southeast Asia, including Brunei Darussalam and in northern Australia. It is estimated that 40% of all cases occur in the East Asia Pacific region [1]. 

Brunei Darussalam is a country in Southeast Asia, located in the northeastern part of Borneo island, with geographical coordinates of 4°30′ N 114°40′ E. The country is divided into four districts: Brunei Muara, Tutong, Kuala Belait, and Temburong. The climate in Brunei Darussalam is tropical equatorial and humid subtropical at higher altitudes with heavy rainfall [2]. The annual average rainfall is more than 2300 mm for the whole of Brunei Darussalam and increases inland, with altitude, to more than 4000 mm with maximum rainfall in the Temburong district [3]. The mean monthly rainfall follows seasonal patterns with two maxima and two minima. The first maximum is from October to January, with December being the wettest month, while the second minor maximum is from May to July, with May being relatively wetter [4]. 

In line with the aims of this special issue on melioidosis, this article addresses various aspects of the disease in Brunei Darussalam. It is divided into the following parts: (i) a review of available literature on melioidosis from Brunei Darussalam; (ii) the current approaches to diagnosis, management, and prevention; (iii) epidemiological study of melioidosis with data obtained from 2015–2016; (iv) melioidosis in animals; and (v) future challenges.

## 2. Review of Literature from Brunei Darussalam

The first published report on melioidosis (24 cases) in Brunei Darussalam was by Luqman et al. [5]. It was more prevalent in the agricultural districts and in the period (1993, 1994) the rate increased from 2.9 to 5.6/100,000. The majority (79.2%) had risk factors, mostly diabetes mellitus. Septicaemia was the presenting feature in 75% of cases, with 62.6% having multi-organ involvement. The mortality rate was 20.8% and on follow-up, 60% of patients presented with relapse of the disease.

The many and varied radiological manifestations of melioidosis in Brunei Darussalam were reviewed by Lim and Chong [6]. Multiple organ involvement, especially the lungs, liver, and spleen, were common. The ‘honeycomb’ appearance of abscesses, especially large abscesses and those seen in the liver, was characteristic of melioidosis. 

Chong et al. reported the characteristics of pancreatic melioidosis (4/65 cases who had undergone CT imaging, median age 29.5 years (range 25–48 years)). Pancreatic involvement ranged from multi-focal micro-abscesses to focal large abscesses, and associated findings included splenic vein thrombosis, peri-pancreatic inflammation, and peri-pancreatic fat streaking. Pancreatic involvement was typically part of multi-organ involvement [7].

Pande and Hj Abdul Kadir reported their experience with melioidosis affecting the extremities (*n =* 14/48 (29.1%); median age 45 years (range 14–55)). The majority (*n =* 13, 92.8%) were men, with 35.7% (*n =* 5) being expatriates working as labourers. Septic arthritis was the most common presentation (*n =* 5), followed by cellulitis, abscess, and osteomyelitis (*n =* 3 each) [8].

The antibiograms of *Burkholderia pseudomallei* from Brunei Darussalam over a period of ten years (679 isolates from 623 patients) showed that carbapenems, third-generation cephalosporins (ceftazidime), piperacillin, and chloramphenicol had the highest susceptibility rates of between 98% and 100%. Amoxicillin-clavulanic acid had a moderate susceptibility rate (76–100%) and quinolones (ciprofloxacin, 32–68%) and co-trimoxazole (6–54%) had the lowest susceptibility rates. It is, however, recognized that the disk diffusion method overestimates the resistance rate for co-trimoxazole [9]. The gentamicin susceptibility of the isolates was not reported.

A case of right frontal lobe brain abscess due to co-infection with *B. pseudomallei* and *Cryptococcus neoformans* in a patient with underlying systemic lupus erythematosus was reported by Samad et al. [10]. The patient was non-diabetic but was on steroid therapy and the diagnosis was confirmed only after surgical drainage.

Chong et al. reported their experience with urogenital melioidosis (*n =* 13, nine new cases, four relapses; median age 38 (range 29–63 years)). The kidneys were involved in 72.3% followed by the prostate (60%). Testicular and seminal vesicle involvement was noted in one case each. There was no involvement of the gynaecological system [11]. 

In the clinical case series mentioned above, a male preponderance was noted, with diabetes mellitus being the most common risk factor [7,8,11]. 

## 3. Current Approach to Diagnosis, Management, and Prevention

### 3.1. Microbiological Diagnosis

In Brunei Darussalam, the diagnosis of melioidosis is confirmed by the isolation of *B. pseudomallei* from clinical specimens, such as blood, sputum, abscess aspirates, pericardial fluid, skin lesions, or other clinical specimens. The clinical specimens are collected during the initial admission to the hospital. Positive blood culture bottles are subcultured onto blood agar and MacConkey agar; urine specimens are inoculated onto cystine lactose electrolyte-deficient (CLED) agar; respiratory specimens, onto blood agar, MacConkey agar, and chocolate agar; and pus specimens into cooked meat broth. These are incubated at 37 °C for at least 24 h before being examined. For pus specimens, subsequent subcultures are made from the broth onto blood agar and MacConkey agar and reincubated at 37 °C for at least another 24 h before re-examination. Selective agars, such as those studied by Peacock et al. [12], are not used in Brunei Darussalam, which means that in some cases growth of *B. pseudomallei* may be missed. In a patient with high clinical suspicion of melioidosis, but with negative cultures, reactivity in a *B. pseudomallei* serological test (sent abroad) is taken as a strong evidence of the disease.

The cultures are then examined for colony morphology and Gram-staining. Morphologically, *B. pseudomallei is* identified as large, wrinkled colonies that have a metallic appearance with an earthy odour. On Gram staining, the organism appears as Gram-negative bacilli with bipolar staining, which gives a characteristic ‘safety pin’ appearance. The cultured colonies are further identified using the VITEK system, a commercial bacterial identification system. However, it is noted that with the use of the VITEK system there is potential for misidentification of *B. pseudomallei* as *Burkholderia cepacia*, which is regionally dependent [13]. This is of particular significance as the study reporting this was from Malaysian Borneo, on the same island as Brunei Darussalam.

All of the isolated organisms are tested for antibiotic susceptibility using Etest and, at present, the results of susceptibility testing are not validated by an external reference laboratory.

### 3.2. Management

Patients who are clinically suspected to have melioidosis, particularly those with a background history of uncontrolled diabetes mellitus, are empirically treated with an antibiotic that has activity against *B. pseudomallei*, i.e., either with intravenous (IV) ceftazidime (up to 2 g every 8 h) or, for clinically-severe cases, with IV imipenem (up to 1 g every 8 h) or IV meropenem (up to 1 g every 8 h) while waiting for culture results to become available.

In patients with confirmed *B. pseudomallei* bacteraemia, and in those patients who were empirically treated with either IV imipenem or meropenem and who have been stable for 48 h, the IV antibiotic is de-escalated to IV ceftazidime. Further investigations, like chest radiographs, ultrasound of the abdomen and pelvis, or computed tomography (CT) scan of the thorax, abdomen, and pelvis (CT-TAP), are usually performed to exclude deep-seated infection with organ abscesses that may be drainable.

The antibiotic treatment for melioidosis comprises two phases, i.e., the intensive phase and the eradication phase. In the intensive phase, IV antimicrobial therapy with either IV ceftazidime or IV meropenem or imipenem is recommended for at least 10–14 days for pulmonary disease or if there are no other obvious deep-seated sources. The intensive phase may be extended to at least 4–6 weeks with drainage of deep-seated abscesses. The patient is then continued on to the eradication phase with oral co-trimoxazole (5 mg/kg of the trimethoprim component every 12 h) as the first line antimicrobial therapy for at least 12 weeks in total. If co-trimoxazole is contraindicated or the patient develops adverse reactions to the medication, then the second-line treatment would be with oral co-amoxiclav (625 mg every 8 h) and oral doxycycline (2 mg/kg every 12 h) to complete a total minimum of 12 weeks. However, clinicians need to be aware of the risk of treatment failure with this second-line eradication phase regimen [14,15]. Currently no post-exposure prophylaxis is offered in Brunei Darussalam.

### 3.3. Preventive Measures

Patients who are admitted with suspected or confirmed pulmonary melioidosis are nursed in droplet isolation for at least 24 h after appropriate antibiotics are commenced. 

In the laboratory setting, biosafety level 3 practices are adopted while handling and processing specimens [16].

## 4. Epidemiological Study of Melioidosis with Data Obtained from 2015–2016

### 4.1. Material and Methods

All culture-positive cases of melioidosis recorded between January 2015 and December 2016 were retrieved from the electronic database of the Clinical Microbiology Laboratory, Department of Laboratory Services, RIPAS Hospital. All patients were investigated and managed as per the protocol mentioned in the previous section.

Demographic, clinical and diagnostic information (laboratory and imaging) was collected through the Hospital Information Management System (BruHIMS). The study was approved by the Medical and Health Research and Ethics Committee (MHREC).

Data are presented as descriptive statistics. Wherever possible, differences between groups were analysed using the chi-square test. A *p*-value of <0.05 was considered significant. 

### 4.2. Results

#### 4.2.1. Demographics

A total of 115 cases were detected in 2015 (*n =* 46) and 2016 (*n =* 69). There were 84 (73%) male and 31 (27%) female patients with an age range from 2 to 86 years (median 48 years).

There were four (3.5%) patients under the age of 15 years, all with no risk factors, who presented with abscesses in soft tissue (*n =* 3) and neck (*n =* 1). They were admitted in the months of January, April, August, and December.

Excluding a two-year-old expatriate patient, the median age of expatriate patients was 41.5 years (*n =* 25). The age range for Bruneian patients (*n =* 89) was 14 to 86 years with a median age of 52 years.

The ethnic distribution of the cases is given in Table 1.

During 2015–2016, the ethnic distribution of the population of Brunei Darussalam was Malay (65.7%), Chinese (10.3%), and expatriates (23.9%) [17].

The overall incidence rates per 100,000 population in 2015 and 2016 were 11 and 16.3, respectively. 

The number of cases and incidence rate per 100,000 population from each of the four districts is presented in Table 2.

Population figures obtained from http://www.depd.gov.bn/sitePages/Population.aspx [17].

**Figure 1:**
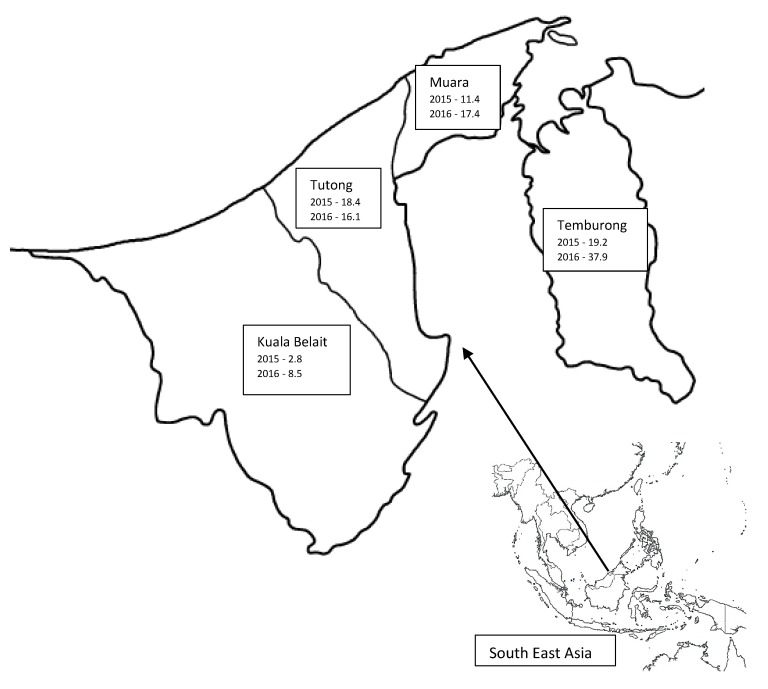
Map showing incidence/100,000 population for 2015 and 2016 by district, Brunei Darussalam.

The incidence was highest in the Temburong district in both years (Figure 1). Except in the Tutong district, there was an increase in incidence for the remaining districts from 2015 to 2016.

#### 4.2.2. Month-Wise Number of New Cases

**Figure 2:**
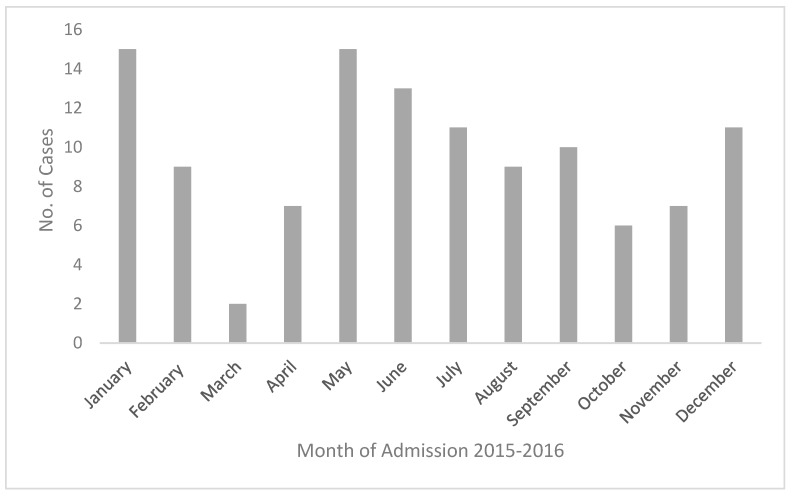
Meliodosis cases by month (2015–2016).

The number of cases (2015 and 2016 combined) according to the month of admission is presented in Figure 2.

The maximum number of admissions were recorded in the months of January and May with smaller peaks in June, July, and December.

#### 4.2.3. Risk Factors

A number of risk factors were identified and are presented in Table 3.

Diabetes mellitus was the most common risk factor, recorded in 74.8% of cases. More than one risk factor was present in 37.4% of cases, and there was no obvious risk factor seen in 7.8% (*n =* 9). 

#### 4.2.4. Clinical Presentations and Diagnosis

Various clinical presentations were recorded (Table 4), pneumonia being the most common (47%). In seven cases (6%) no focus of infection could be found.

Thirteen of 25 cases of septic shock had pneumonia as the primary source of infection, including in the 54 cases of pneumonia.

Internal organ abscesses were most commonly noted in spleen and liver (Table 5).

In most cases (67%), the diagnosis was confirmed by blood culture (Table 6).

CT-TAP was performed in 50.5% (*n =* 58) of cases. The reasons for a patient not undergoing CT-TAP were septic shock and serious illness, presentation as cellulitis or soft tissue abscess, and issues with payment for expatriate workers.

#### 4.2.5. Outcome of Treatment

In the years 2015 and 2016, there was an overall mortality of 27% (Table 7). Mortality in patients presenting with septic shock was 64%. The mortality in women was 41.9% (13/31) compared to 21.4% (18/84) in men (*p* < 0.005). 

Of the five cases with relapse, one patient died during readmission with septic shock (not included in the 31 fatalities).

In 2015, 11 patients (23.9%) were admitted with septic shock and the mortality was 26.1% (*n =* 12) compared to 14 patients (20%) and 27.5% (*n =* 19), respectively, in 2016. Five of the nine patients without any risk factors succumbed to the disease.

### 4.3. Discussion

This is the first detailed epidemiological study of melioidosis from Brunei Darussalam. The data have revealed a slight increase in incidence from 11 to 16 per 100,000 population between 2015 and 2016. It also showed a high number of expatriate workers being affected. Diabetes mellitus and chronic renal disease were the most common risk factors, whilst pneumonia and soft tissue abscess were the most common clinical presentations. The data suggested a possible link with seasonal variation in rainfall with more cases diagnosed in the wet months. Interestingly the highest incidence rate was from the Temburong district, which has the highest annual rainfall. The mortality was 27%, being higher in women and patients presenting with septic shock.

For the purpose of discussion, we have compared our results with other large epidemiological studies from Asia-Pacific published after 2010 [18,19,20,21,22,23,24,25]. Our findings of male preponderance, the median age of patients, diabetes mellitus and chronic renal disease as the most common risk factors, and pneumonia and soft tissue abscesses as the most common presentations, are similar to the studies reviewed.

Though occupational history was not consistently recorded in the hospital notes, similar to a previous study [8], 25 expatriate patients with varied nationalities (median age 41.5 years) were noted. Generally the majority of these were labourers working in the agriculture or construction industries, with a high chance of exposure to contaminated soil and water.

The overall incidences of 11 and 16.3 per 100,000 population in 2015 and 2016, respectively, are considerably higher than those reported from Thailand and Singapore [22,25]. It is important to note that, in Singapore, a recent decrease in incidence was reported [25], while the incidence has remained constant or increased in Australia and Thailand [18,22]. Our rates are comparable to the rate of 16.45/100,000 reported from the state of Kedah, Malaysia by Hassan et al. [21]. In contrast, another study based on the melioidosis registry from the Pahang state of Malaysia reported a much lower rate of 4.3/100,000 (adult 6.0/100,000 and paediatric 1.6/100,000) [26]. This reflects geographical differences, as seen in our study, with different rates between districts, even for a small country.

The current rates reported showed marked increase compared to the incidence rates (2.9 to 5.6/100,000 for 1993 and 1994, respectively) reported by Luqman et al. [5]. The upsurge may well be contributed to by better awareness and improvement in diagnostic facilities. Melioidosis was possibly under-diagnosed and under-reported in the past [27]. Another plausible reason for the upward trend is the rise in the overall population along with an increase in the number of people with risk factors, such as diabetes and chronic kidney disease. 

There were four patients (3.5%) under the age of 15 years, comparable to the rate reported by Hassan et al., (5.5%), and higher than the rate reported by How et al., (1.6/100,000) [21,26]. Consistent with reports in the literature, our paediatric patients had no risk factors, presented with soft tissue abscesses, two of them during the wet months of December and January, and recovered completely with treatment [28]. Unlike Kingsley et al., we did not observe any cases of neonatal melioidosis [29].

In the present study, a higher number of cases was observed in the wet months of May to July and December to February, with 64.3% being admitted during these months. The incidence was also higher in the Temburong district, which records the highest rainfall amongst the four districts. A high level of moisture in the soil during months of high rainfall has been reported to correlate with an increased incidence of melioidosis [30,31]. This association has been reported from Australia [18,20], Singapore [25], Malaysia [21], and India [23]. In contrast, Limmathurotsakul et al. reported a negative association between the total annual rainfall and the number of cases in each year of their study from Thailand [22]. However, in this study monthly rainfall data and the number of cases per month were not available. Consistent with an incubation period of a few weeks, the incidence may also be higher in the corresponding period after high rainfall [32]. 

Diabetes mellitus was recorded as a risk factor in 74.8% of cases, similar to the proportion in India [23] and Malaysia [24]. Other studies have reported it in 39–57% of their cases [18,20,21,22,25]. In two studies from Australia [18,20], excessive alcohol use was the second most common risk factor. In the present study it was noted in 5.2% of cases but could be underreported due to a reluctance to volunteer such a history. However, the sale of alcohol is banned in Brunei Darussalam and, therefore, our finding is likely to be a true reflection of the real situation.

The number of patients with melioidosis without any risk factors was lower (7.8%, *n =* 9) in the present study compared to other studies (13–22%) [18,20,23,24] but similar to that reported by Kingsley et al. [29]. It has been suggested that individuals with no risk factors have less severe symptoms and mortality is rare [29]. However, in the present study 5/9 patients succumbed to the disease, three of whom presented with septic shock, and two with large soft tissue abscesses suggesting fulminant infection.

Consistent with the studies reviewed, pneumonia was the most common clinical presentation followed by soft tissue abscesses [18,20,21,22,23,24,25]. Septic shock was the mode of presentation in 21.7% (*n =* 25) cases. In a review of case reports published from Malaysia, a high frequency of primary neurological presentation was noted (7.5%) [29]. The rates reported by other authors [18,21,23,24] and one case in the present study is much lower. This may be due to selection bias in the case reports reviewed.

Internal organ abscesses were most common in the spleen and liver, as noted in the literature [18,21,23,24]; however, the proportion was much higher in the present study. We had only one case of subdural empyema and no cases with mycotic aneurysms or pericardial involvement. The proportion of prostatic abscesses in the present study was 4.3%; there is a wide variation reported in the literature, from 0.3% in Thailand [33], to a high of 20% in Australia [18]. Our finding of the frequency of parotid involvement (*n =* 1, 0.9%) is comparable to other studies [23,24,29], but much lower than that reported in Thai children (20–30%) [34]. We did not find any case with pancreatic involvement in the present study. The four cases previously reported were seen over a period of six years, compared to two years of the present study [7]. Moreover, only about 50% of patients in the present study underwent CT-TAP for various reasons, which could have resulted in under-reporting of internal organ abscesses.

The overall mortality in the present study was 27%, with a higher mortality in women (41.9%) compared to men (21.4%; *p* < 0.05). This association has also been reported by Kingsley et al. [29]. Mortality was also significantly higher in patients who presented with septic shock.

There is a wide variation in the reported mortality in the literature, from 9.5% in India [23], 42.6% in Thailand [22], and between 32–63% in Malaysia [19,21,24]. Decreasing mortality rates have been reported from Australia [18] and Singapore [25]. The reasons put forward for this are access to standardized health care and the institution of prompt treatment according to recommendations [35]. In Singapore, in particular, it is thought to be due to efforts to optimise diabetes care and enhanced environmental and water management [25]. Our current rate is slightly higher than that reported in the earlier study by Luqman et al., (20.3%) [5], possibly due to late presentations and multiple comorbidities.

Five cases (4.3%) had recurrence of the disease, which is a similar rate to that reported by Currie et al. [18]. One of the 31 cases that did not survive also presented with a recurrence. They had underlying risk factors of diabetes mellitus (4/6), malignancy (2/6), and chronic renal disease (1/6). Non-compliance with previous treatment was recorded in two cases. The institution of prompt treatment along international guidelines explains the lower current rate compared to relapses seen in 60% of cases in the past [5].

The strength of the present study is that, owing to the method of data collection, it can be taken as a true reflection of the disease state in the country. A limitation of the study was its retrospective nature. Data, particularly on occupation and alcohol abuse as risk factors, were not adequately recorded.

## 5. Melioidosis in Animals

All livestock farms in Brunei Darussalam are advised through the Department of Agriculture and Agrifood about good animal husbandry practices as a method of prevention of diseases.

According to the Animal Health and Disease Control Unit under the Livestock and Veterinary Services Division, 93 and 128 goats were treated for presumed melioidosis in 2015 and 2016, respectively. This was based on farmers’ complaints and clinical findings, but no bacteriological evidence was obtained. A positive response to treatment was noted in 80% of cases. The high susceptibility of goats to melioidosis has been reported in the literature [36,37]. 

It has been suggested that goats and humans are exposed to similar levels of *B. pseudomallei* in the environment and, hence, the incidence is likely to be similar [37]. However, data on melioidosis incidence in animals are not available in Brunei Darussalam to confirm this. 

In Brunei Darussalam, the goat farms are mostly small, with herds less than 500 head. The goat houses use raised flooring and practice ‘cut and carry’ grass for feeding to avoid direct contact of animals with the soil. Cattle livestock are imported from Australia, and Sabah and Sarawak in Malaysia, and kept in holding yards for seasonal sales and are then slaughtered shortly afterwards (short-term rearing), so that goats make up the longest-standing ruminant population. Buffaloes are less susceptible to melioidosis, as noted in a review [38].

In late 2016, a few goat and sheep carcasses were submitted for autopsy with lesions suspicious of melioidosis. The farm had recently carried out excavation work, followed by heavy rain. The animals were 2–6 months of age and, hence, thought to be immunocompromised. The bacteriological tests were negative for *B. pseudomallei*. The sick animals from the farm were treated and water was disinfected as a precautionary measure. Workers in the farm and fodder plantation with respiratory symptoms were advised to attend the nearest health centre.

## 6. Future Direction

It is clear from the Health Information Booklet 2016, published by the Ministry of Health, Brunei Darussalam, that diabetes, pneumonia, and septicaemia are amongst the top 10 causes of death in the country [39]. The number of cases of melioidosis in 2016 (*n =* 69) was listed after the number of cases of gastroenteritis and food poisoning in the list of waterborne diseases. However, melioidosis does not feature in the list of notifiable diseases in Brunei Darussalam, unlike in Australia [20] and Singapore [25].

Evidence-based guidelines are now available for prevention of melioidosis in endemic areas [40]. Suntornsut et al. explored the barriers in implementing preventive measures for melioidosis and have suggested a number of interventions. Based on these, the authors have recommended a multifaceted intervention involving community and government agencies [41].

Kingsley et al. have stressed the importance of making melioidosis notifiable and starting registries in endemic countries to improve the efforts to effectively control the disease [29]. Through such registries, it will be possible to obtain reliable clinical and epidemiological information, note trends in the incidence, and determine effective treatment and mortality.

Involvement of the various stakeholders that encounter melioidosis in their practice and the formation of a national registry would help to ensure that data collected is complete and standardized. This will allow comparisons and the analysis of trends. Thus, the formation of a regional or international registry would allow the condition to be better studied. If Brunei Darussalam were to follow Australia and Singapore in making the condition a notifiable disease, this would further enhance awareness and this may have an impact on the clinical course of the disease in the future. 

## Figures and Tables

**Table 1:** Ethnic distribution of cases of melioidosis.

Country	No.	%
Malay	88	76.5
Chinese: Bruneian	1	0.9
*Expatriates*	26	22.6
Bangladesh	10	8.7
Indonesia	8	7
India	4	3.5
Philippines	1	0.9
Thailand	1	0.9
Nepal	1	0.9
Malaysia	1	0.9

**Table 2:** District-wise incidence of melioidosis/100,000 population for 2015–2016.

District	Pop. 2015	Cases	Inc/100,000	Pop. 2016	Cases	Inc/100,000
Muara	288,400	33	11.44	292,705	51	17.42
Tutong	48,700	9	18.48	49,438	8	16.18
Kuala Belait	69,000	2	2.89	69,992	6	8.57
Temburong	10,400	2	19.23	10,543	4	37.93

**Table 3:** Risk Factors for melioidosis.

Risk Factor	Cases	%
Diabetes mellitus	86	74.8
Chronic renal disease	23	20
Cardiac disease	17	14.8
Lung diseases (COPD/TB)	16	13.9
Excessive alcohol intake	6	5.2
Thalassemia	5	4.3
Malignancy	2	1.7
None	9	7.8

Note: Most of the chronic renal disease patients were those with end-stage renal failure on dialysis and thalassemia patients who were transfusion-dependent and had iron overload.

**Table 4:** Clinical presentation of melioidosis.

Presentation	Cases	%
Septic shock	25	21.7
Pneumonia	54	47
Soft tissue abscess	21	18.3
Musculo-skeletal	10	8.7
Neurological	1	0.9
No evidence of primary focus	7	6

**Table 5:** Internal organ abscess in melioidosis.

Organ	Cases	%
Spleen	27	23.5
Liver	20	17.4
Prostate	5	6
Lymph node	4	3.5
Kidney	2	1.7
Parotid	1	0.9
Brain	1	0.9

**Table 6:** Source of culture for diagnosis of melioidosis.

Modality	No. of Isolates	%
Blood culture	77	67
Fluid culture	11	9.6
Pus culture	37	32.2

**Table 7:** Outcome of treatment of cases.

Outcome	Cases	%
Recovery	79	68.7
Death	31	27
Relapse	5	4.3
